# Mechanism of Action of Cyanidin 3-O-Glucoside in Gluconeogenesis and Oxidative Stress-Induced Cancer Cell Senescence

**DOI:** 10.3390/antiox11040749

**Published:** 2022-04-09

**Authors:** Yaoyao Jia, Chunyan Wu, Adriana Rivera-Piza, Yeon-Ji Kim, Ji Hae Lee, Sung-Joon Lee

**Affiliations:** 1 Department of Biotechnology, College of Life Science and Biotechnology, Korea University, Seoul 02841, Korea; janciey0227@gmail.com (Y.J.); chunyanwu87@gmail.com (C.W.); adri.ripiz@gmail.com (A.R.-P.); mindfulless@hanmail.net (Y.-J.K.); jihae@korea.kr (J.H.L.); 2 Department of Food Bioscience and Technology, College of Life Sciences and Biotechnology, Korea University, Seoul 02841, Korea

**Keywords:** Cyanidin 3-O-glucoside, natural products, phytochemicals, antioxidants, adiponectin signaling, hepatic autophagy, oxidative stress, anti-carcinogenic activity, HepG2 cells

## Abstract

Cyanidin-3-O-glucoside (C3G) is a natural anthocyanin abundant in fruits and vegetables that interacts and possibly modulates energy metabolism and oxidative stress. This study investigated the effect of C3G on gluconeogenesis and cancer cell senescence. C3G activates adenosine monophosphate-activated protein kinase (AMPK), a cellular energy sensor involved in metabolism and the aging process. C3G suppressed hepatic gluconeogenesis by reducing the expression of gluconeogenic genes through the phosphorylation inactivation of CRTC2 and HDAC5 coactivators via AMPK. C3G did not directly interact with AMPK but, instead, activated AMPK through the adiponectin receptor signaling pathway, as demonstrated through adiponectin receptor gene knockdown experiments. In addition, C3G increased cellular AMP levels in cultured hepatocytes, and the oral administration of C3G in mice elevated their plasma adiponectin concentrations. These effects collectively contribute to the activation of AMPK. In addition, C3G showed potent antioxidant activity and induced cellular senescence, and apoptosis in oxidative-stress induced senescence in hepatocarcinoma cells. C3G increased senescence-associated β-galactosidase expression, while increasing the expression levels of P16, P21 and P53, key markers of cellular senescence. These findings demonstrate that anthocyanin C3G achieves hypoglycemic effects via AMPK activation and the subsequent suppression of gluconeogenesis and exhibits anti-cancer activity through the induction of apoptosis and cellular senescence.

## 1. Introduction

Obesity is an epidemic disease worldwide that leads to the development of type 2 diabetes mellitus and various cancers. Oxidative stress due to high plasma glucose levels contributes to pathological changes associated with diabetes and its complications, as well as senescence and cancers [1]. Reactive oxygen species (ROS) have a dual role in cancer [2]. First, ROS can promote pro-tumorigenic signaling, facilitating cancer cell proliferation, survival and adaptation to hypoxia. Second, ROS can promote antitumorigenic signaling and trigger oxidative stress-induced cancer cell death [3]. Depending on the ROS concentration, molecular species and subcellular localization, cell components and signaling pathways are affected positively or negatively [4]. Cause–effect relationships between cellular ROS and cell senescence have been investigated through diverse pathways [5,6,7] and hence are relevant for therapeutic interventions, which aim to modulate ROS levels in cancer cells, as well as in aging processes [4,8], e.g., low ROS levels improve the defense mechanisms by inducing adaptive responses, which contributes to stress resistance and longevity, while high ROS levels induce insufficient adaptive responses, which may contribute to aging onset and progression [4,9,10].

Thus, the removal of ROS has been suggested for the prevention of metabolic disorders as well as conditions such as senescence and carcinogenesis related to aging [1,11]. Plant-based compound therapies, which have negligible adverse effects, are promising options for the treatment of obesity-related diseases, such as type 2 diabetes and cancers. Particularly, these natural compounds have been shown to induce the appearance of cellular senescence characteristics in cancer cells as well as to delay cellular senescence in normal primary cells [9]. Additionally, cellular oxidant/antioxidant balance is influenced by dietary agents, such as flavonoids, which may also act as pro-oxidants and induce apoptosis in cancer cells [12,13]. Therefore, ROS may modulate the tumor suppression process, which is induced by senescence, thus participating in anticancer mechanisms, although ROS may act as tumor promoters in definite conditions [4,14,15,16].

AMP-dependent kinase (AMPK) is an energy sensor with multiple effects on cellular metabolism and aging. It functions antagonistically towards mTORC1 to induce the autophagy pathway [17]. AMPK activation can increase lifespan and healthspan due to its relationships with mTORC1 and autophagy. Genetically modified mice with low mTORC1 levels but normal mTORC2 levels had healthy lifespans [18], while mice deficient in S6K1 (mTORC1 effector) were reported to have longer lifespans [19].

Natural small molecules and dietary compounds have demonstrated benefits for the treatment of various metabolic disorders. The biological effects of these natural compounds tend to involve more than one pharmacological target or site of action, a trait known as pleiotropism [20,21]. Anthocyanins, commonly found in cereals, roots, vegetables and fruits that render them vivid red to blue are a subcategory of flavonoids that also belong to polyphenol compounds and have been suggested as potent natural compounds with several biological activities [21,22,23]. Currently, more than 500 different anthocyanins, varying from the presence/position of aliphatic or aromatic carboxylates to the glycosylation and acylation pattern, have been reported [23,24]. Interest in anthocyanin pigments has drastically intensified due to their potential health-promoting benefits, such as anti-inflammatory and anti-carcinogenic activity, obesity control and diabetes alleviation [20,21,22,23,24,25]. For example, the anti-diabetic effects of anthocyanins have been well documented by inducing an anti-hyperglycemic effect via the protection of beta-cells, thereby improving insulin secretion and resistance, enhancing liver function and inhibiting carbohydrate-hydrolyzing enzymes [25,26]. Nevertheless, none of these studies have clearly reported the potential mechanisms of action and molecular targeting of anthocyanins, and thus, they remain elusive.

Among the most common anthocyanins, cyanidin-3-O-β-glucoside (C3G) has the highest antioxidant activity and confers a promising protective effect against oxidative stress-induced damage, as shown using in vivo models [22,23]. It possesses a C6–C3–C6 carbon backbone, wherein two aromatic rings are joined with a heterocyclic aromatic ring. C3G is a more potent antioxidative compound than the other six molecules belonging to its class. C3G has antioxidative and anticancer effects and can suppress hyperglycemia-induced oxidative stress in diabetes [22,25,26,27]. Numerous studies have established that anthocyanins show potential anti-cancer effects on human health since can regulate oxidant and antioxidant balance, thereby inducing apoptosis, particularly in cancer cells [12,13,14,22,26], and highly recommended anthocyanins as nutraceuticals for cancer prevention and management [22].

In this study, we investigated the molecular mechanism through which C3G exerts hypoglycemic effects via AMPK activation and anti-cancer effects via the induction of apoptosis and cellular senescence. C3G induces phosphorylation and, therefore, the activation of AMPK, while suppressing hepatic gluconeogenesis through the inhibition of gluconeogenic gene expression. C3G showed potent antioxidative effects, leading to cellular senescence and apoptosis.

## 2. Materials and Methods

Commercially available materials and reagents used in this study are listed in Supplementary Table S1.

### 2.1. Cell Culture and Mouse Experiments

The human hepatocellular carcinoma cell line (HepG2) and human embryonic kidney cell line (HEK293) were acquired from the Korean Cell Line Bank (Seoul, Korea). These cells were cultured in Dulbecco’s modified Eagle’s medium (DMEM) supplemented with 10% fetal bovine serum (FBS), 1% penicillin/streptomycin (PEST) and incubated at 37 °C under a humidified 5% *v*/*v* CO_2_. For animal procedures, C57BL/6J male mice were obtained from Samtako Co. (Kyunggido, Korea), while PPARα-deficient mice were obtained from Taconic (Hudson, NY, USA). All mice were housed in a specific pathogen-free environment, and the mice were randomly assigned to two groups: high-fat diet (HFD)-fed with vehicle (phosphate-buffered saline, PBS) and HFD-fed with oral administration of C3G (100 mg/kg body weight) for 8 weeks of treatment. Body weight and food intake were assessed weekly. In the study of AMPK phosphorylation and autophagy markers, LC3-I and II fenofibrate (FF, 100 mg/kg body weight) groups were assessed in addition to the vehicle and C3G groups. At the end of the experimental period, the mice were fasted for 12 h and sacrificed. Blood was collected retro-orbitally or by cardiac puncture in tubes containing ethylenediaminetetraacetic acid (EDTA; BD) and centrifuged at 2000 rpm (Labogene Co., mod. 1248R, Lillerød, Denmark) and 4 °C for 30 min to collect plasma, and then samples were kept at −80 °C. Mice livers, white adipose tissues (epididymal, visceral and perirectal fat) and brown adipose were collected and snap-frozen in liquid nitrogen and then stored at −80 °C for further use. All animal procedures conducted in this study were approved by the Animal Experiment Committee of the university (Protocol No. KUIACUC-20090420-4).

### 2.2. Measurement of the Rate of Gluconeogenesis

HepG2 cells were seeded in DMEM (containing 4500 mg/L glucose and phenol red) (Life Science, Seoul, Korea) with 10% FBS and 1% PEST at a density of 10^6^ cells/well in a 6-well plate for 24 h, and the medium was changed to assay medium (DMEM without glucose, phenol red and FBS), to which either C3G (10 and 50 μM) was added or metformin was added as a positive control, and treated with or without the compound C (AMPK inhibitor) for another 24 h. Cells were washed 2 times with 1× PBS; then, DMEM containing 20 mM sodium lactate, 2 mM sodium pyruvate, 2 mM L-glutamine and 15 mM HEPES (4-(2-hydroxyethyl)-1-piperazineethanesulfonic acid) was added to the cells; and 100 μM of 8-(4-Chlorophenylthio)adenosine 3′,5′-cyclic monophosphate sodium salt (8-CPT-cAMP) was added as the control for 6 h. The culture medium was collected, and glucose concentrations were determined using an automated clinical chemistry analyzer (Cobas111, Roche, Basel, Switzerland) according to the manufacturer’s instructions.

### 2.3. Autophagy Analysis

Autophagy staining was measured using an autophagy and cytotoxicity dual-staining kit (Cayman, MI, USA) according to the manufacturer’s instructions. Briefly, HepG2 cells were seeded at 5 × 10^4^ cells/well for 24 h and treated with C3G (10 or 50 μM). The cells were stained with 100 μL of monodansylcadaverine (MDC) solution for 10 min at 37 °C. After washing with cell-based buffer, autophagic vacuoles stained with MDC were detected with a Zeiss LSM 5 Exciter confocal microscope (100×/1.30 oil differential interference contrast) and processed using Zeiss LSM510 v.3.2 software (Carl Zeiss, Jena, Germany). Hepatic autophagy was analyzed through transmission electron microscopy (TEM). In these experiments, HepG2 cells were treated with C3G (10 or 50 μM) for 12 h, fixed with 2% *w*/*v* formaldehyde and 0.1 M sodium cacodylate, and then stored at 4 °C overnight. The cells were postfixed with 2% osmium tetroxide and stained with 0.5% *w*/*v* uranyl acetate for 30 min, followed by an increasing gradient dehydration step using ethanol and propylene oxide. Then, infiltration with Spurr’s resin was performed overnight, and images were examined at 80 kV with a JEM1010 electron microscope (JEOL, Tokyo, Japan).

### 2.4. In Vitro AMPK Activity Assay

An in vitro AMPK activity assay was performed with the AMPK Kinase Enzyme System (Promega, Madison, WI, USA) according to the manufacturer’s instructions. Briefly, C3G was incubated with reaction solution containing SAMS peptide and AMPK (α1/β1/γ1 or α2/β1/γ1) with or without A-769662 to examine the allosteric binding site. The reaction was initiated with the addition of ATP (150 μM) and, after incubation at room temperature for 1 h, the reaction was terminated through the addition of ADP-Glo^TM^ Reagent. Kinase detection reagent was added to detect the ADP product, and AMPK activity was quantified using a luminescence meter (Victor 2, Perkin Elmer, Norwalk, CT, USA).

### 2.5. Gene Knockdown with AMPKα and Adiponectin Receptor 1/2 siRNA

HepG2 cells were seeded in 12-well plates at a density of 2×10^5^ cells/well, and when the cells were 50–60% confluent, they were transfected with AMPKα and adiponectin receptor 1/2 siRNA duplex (4 μL/well; Santa Cruz, CA, USA) for 24 h using Lipofectamine 2000 reagent (Promega, Madison, WI, USA). After transfection, the cells were treated with C3G (10 and 50 μM) or A-769662 (100 μM) as the positive control and 0.1% *v*/*v* dimethyl sulfoxide (DMSO) as the vehicle for 24 h. The protein levels of AMPKα were analyzed through immunoblotting to confirm knockdown efficiency.

### 2.6. Immunoblotting

Total protein was extracted from livers and measured through immunoblotting, as described previously [28]. After protein extraction, immunoblotting was performed using RIPA buffer (10 mM Tris-HCl, pH 7.5, 1% NP-40, 0.1% *w*/*v* sodium deoxycholate, 0.1% *w*/*v* sodium dodecyl sulfate (SDS), 150 mM NaCl and 1 mM EDTA) containing 1% *v*/*v* protease and phosphatase inhibitor cocktail, as described previously. The protein concentrations were assessed using Bio-Rad reagent (Bio-Rad, Hercules, CA, USA). The proteins were denatured and run on an SDS polyacrylamide gel electrophoresis (PAGE) gel, and immunoblotting was performed as described previously. The proteins separated through SDS-PAGE were transferred to a nitrocellulose membrane (Schleicher and Schuell Bioscience, Dassel, Germany). Nonspecific binding was blocked using 5% *w*/*v* nonfat dry milk in Tris-buffered saline containing Tween (TBS-T) buffer for 1.5 h at room temperature. The membranes were incubated with primary antibody overnight at 4 °C, washed with TBS-T buffer and further incubated with secondary antibodies for 1 h at room temperature. Immunoblot images were obtained with the ChemiDoc^TM^ XRS+ imaging system (Bio-Rad, Hercules, CA, USA), and protein contents were quantified with Gel-Pro Analyzer software. Protein levels were normalized to α-tubulin or β-actin.

### 2.7. Total RNA Extraction and Quantitative Polymerase Chain Reaction (qPCR)

Total RNA was isolated from liver tissue using RNAiso Plus reagent following the manufacturer’s protocol (TaKaRa Bio Inc., Otsu, Japan). cDNA was synthesized using the Rever Trace^®^ RT Master Mix Kit (Toyobo, Osaka, Japan). To synthesize cDNA, 1 μg of total RNA was mixed with the “4× DN Master Mix” (a buffer solution containing RNase inhibitor) and gDNA remover and then incubated at 37 °C for 15 min. Then, “5× master mix” containing the highly efficient reverse transcriptase “Rever Tra Ace^®^”, RNase inhibitor, oligo dT primers, random primers and dNTPs was added to the mixture, which was incubated at 50 °C for 5 min and then at 98 °C for 5 min. The levels of mRNA expression were assessed using the iQ5 Real-Time PCR Detection System (Bio-Rad, Hercules, CA, USA) and THUNDERBIRD^TM^ SYBR^®^ qPCR Mix. Relative levels of gene expression were calculated through the threshold cycle (CT) method and normalized to cyclophilin or L32 expression.

### 2.8. Intracellular Calcium Assay

HepG2 cells were seeded overnight in 96-well plates at a density of 4 × 10^4^/well. An equal volume of 2× the loading solution Fluo-4 Direct™ calcium reagent (Invitrogen, Carlsbad, CA, USA) was then added to the wells, and the plates were incubated at 37 °C for 30 min. Next, the cells were treated with 10 μM of A-23187 (a calcium ionophore, as a positive control) or 10 or 50 μM of C3G, and the calcium flux was measured with a Victor X2 plate reader (PerkinElmer, Santa Clara, CA, USA) at 485/520 nm excitation/emission wavelengths according to the manufacturer’s protocol (Invitrogen, Carlsbad, CA, USA).

### 2.9. Reporter Gene (Luciferase) Assay

HepG2 cells were seeded overnight in 24-well plates at a density of 2 × 10^4^ cells/well. The next day, using Lipofectamine 2000 reagent (Invitrogen) and following the manufacturer’s instructions, the cAMP response element (CRE) or FoxO1 firefly luciferase reporter or a mock vector were co-transiently transfected with Renilla expression vector. At 18 h post-transfection, cells were stimulated with C3G (10 and 50 µM) for 24 h. Then, cells were washed with 150 μL phosphate-buffered saline (PBS) per well followed by cell lysis using 40 or 60 μL of Passive Lysis Buffer (PLB) per well (Promega, Madison, WI, USA). Luciferase activities were measured using a Dual-Glo Luciferase Assay kit (Promega). All luciferase emission measurements were quantified using a Victor X2 plate reader (PerkinElmer, Santa Clara, CA, USA). All firefly luminescence signal were normalized to that of Renilla luciferase driven by the constitutively active SV40 promoter (pRL-SV40; Promega).

### 2.10. Intracellular Reactive Oxygen Species (ROS) Measurement

ROS assessment was conducted using the Fluorometric intracellular ROS kit (Sigma Aldrich) following the manufacturer’s instructions. Briefly, HepG2 cells were seeded in 96-well plates (2 × 10^4^) for 24 h. After, the cells were co-treated with H_2_O_2_ containing 0.1% *v*/*v* DMSO (vehicle), with 10 μM or 50 μM C3G. Fluorescence was measured after 1 h incubation with a microplate reader at 520/605 nm excitation/emission wavelengths.

### 2.11. Caspase-3 Activity Assay

The measurement of caspase-3 activity was conducted with the colorimetric Caspase-3 Assay Kit (Sigma Aldrich, St. Louis, MO, USA) following the manufacturer’s protocol. HepG2 cells were seeded in 96-well plates (1 × 10^5^ cells/well) and co-treated with H_2_O_2_ containing the tested compounds: 0.1% *v*/*v* DMSO (vehicle), 10 μM or 50 μM C3G for 24 h. Fluorescence was measured with a microplate reader at 340/440 nm excitation/emission wavelengths.

### 2.12. Cell Viability Assay

Cell viability was determined through a modified MTT (3-[4,5-dimethylthiazol-2-yl]-2,5 diphenyl tetrazolium bromide) assay. The MTT assay was performed to detect the effect of hydrogen peroxide (H_2_O_2_) and C3G on cell viability. First, HepG2 cells (2 × 10^4^ cells/well) were seeded in 96-well plates for 24 h and treated with various concentrations of H_2_O_2_ for 24 h. After changing the culture medium, 5 mg/mL MTT reagent was added, and the cells were incubated for 4 h (data not shown). Similarly, HepG2 cells (2 × 10^4^ cells/well) were seeded in 96-well plates for 24 h. Cells were exposed to various H_2_O_2_ concentrations and co-treated with 0.1% *v*/*v* DMSO (vehicle), 10 μM or 50μM C3G for 24 h to assess the impact on cell viability. Cell viability was calculated from absorbance at 570 nm measured using a microplate reader.

### 2.13. Senescence-Associated (SA)-β-Galactosidase Assay

HepG2 cells were cultured in 24-well sterile culture plates at a density of 2 × 10^4^ cells/well. The following day, cells were co-treated with H_2_O_2_ containing the tested compounds for 3 days, with medium changed daily. On day 5, the culture medium was discarded, and the cells were fixed with 0.8 mL β-gal staining solution for 15 min. The cells were incubated with 0.8 mL SA-β-gal staining working fluid. A minimum of 450 cells were observed through optical microscopy, and the percentage of positively stained cells was calculated.

### 2.14. Statistical Analysis

All data are shown as the mean ± standard error of the mean (SEM), Tukey’s multiple comparison test and one-way analysis of variance (ANOVA) were used to calculate the significance of differences between each pair of groups using GraphPad Prism version 8.0.0 for Windows, GraphPad Software (San Diego, CA, USA). A value of *p* < 0.05 was considered significant. For metabolomic analysis, ANOVA was performed using SPSS software (Version 12.0, Chicago, IL, USA) to assess significant differences in metabolites in plasma samples from mice fed C3G with an HFD. Duncan’s multirange test was used with the level of significance set to *p* < 0.05.

## 3. Results

### 3.1. C3G Activates AMPK Phosphorylation and Suppresses the Rate of Gluconeogenesis

**Figure 1:**
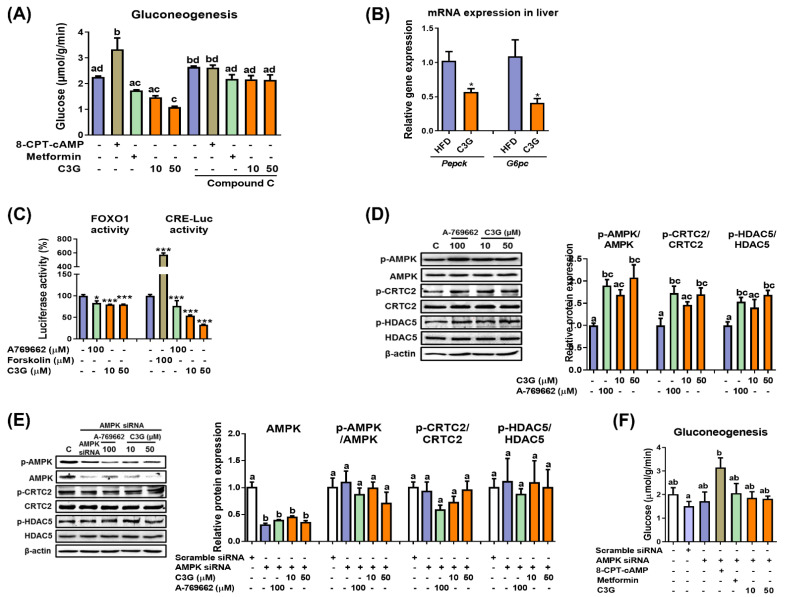
C3G activates AMPK and reduces gluconeogenesis through suppression of gluconeogenic gene expression. (**A**) Rate of gluconeogenesis in HepG2 cells incubated with C3G. 8-CPT-cAMP is a cAMP analogue. Metformin is an AMPK activator, while compound C is an AMPK inhibitor. (**B**) mRNA expression of gluconeogenic genes measured through qPCR. Transcription factor activity assay. (**C**) The activities of FoxO1 and CRE were measured using a luciferase assay. A769662 is an AMPK activator, and forskolin is an adenylate cyclase activator. (**D**) C3G stimulates AMPK, CRTC2 and HDAC phosphorylation in HepG2 cells. (**E**) AMPK, CRTC2 and HDAC5 phosphorylation in HepG2 cells with AMPKα1 gene knockdown. (**F**) Rate of gluconeogenesis in HepG2 cells following AMPKα1/α2 gene knockdown. For comparisons between two groups, Student’s *t*-test was performed. *, *p* < 0.05; ***, *p* < 0.005 compared with controls. For multiple-group comparisons, one-way ANOVA was performed. Different letters indicate statistically significant differences at *p* < 0.05.

In mice, C3G was found to improve hyperglycemia and insulin sensitivity [27]. It was reported that the oral administration of C3G reduced lipid accumulation in the liver and improved glucose tolerance [27]. In this study, we further quantified hepatic ATP and AMP levels, and the results revealed that the AMP-to-ATP ratio was elevated in the livers of mice fed C3G (Supplementary Table S2). An increase in the cellular AMP-to-ATP ratio leads to the activation of AMPK. AMPK activation suppresses hepatic gluconeogenesis. Thus, we next investigated whether C3G affects gluconeogenesis [29]. In HepG2 cells, C3G inhibited the rate of gluconeogenesis similarly to metformin (a positive control), while 8-CPT-cAMP, a cAMP analogue, increased the rate of gluconeogenesis, as expected (Figure 1A).

Next, the rate of gluconeogenesis was assessed when metformin or C3G was incubated with compound C, an AMPK inhibitor (Figure 1A). The results demonstrated that compound C ameliorated the effect of C3G on gluconeogenesis, indicating that C3G suppresses gluconeogenesis in an AMPK-dependent manner. 8-CPT-cAMP and compound C did not show a synergistic effect on gluconeogenesis.

The effects of C3G on gluconeogenesis gene expression were investigated in mouse livers. The mRNA expression levels of *Pepck* (phosphoenolpyruvate carboxykinase) and *G6pc* (Glucose-6 phosphatase), key genes in gluconeogenesis, were significantly reduced in the livers of mice fed C3G (Figure 1B). *Pepck* and *G6pc* expression levels were stimulated by the transcription factors FOXO1 and CREB. Therefore, we next investigated whether C3G affects FOXO1 and CREB transactivation activity using reporter gene assays. The results indicated that C3G suppressed the transactivation of both FOXO1 and CREB, with CREB suppressed to a greater degree than FOXO1 (Figure 1C). The activities of FOXO1 and CREB are stimulated by their coactivators, HDAC5 and CRTC2, respectively. The phosphorylation of CRTC2 and HDAC5 by AMPK inhibits the nuclear localization and activation of CREB and FOXO, respectively. Therefore, we next assessed the phosphorylation of HDAC5 and CRTC2 through immunoblotting assays. The results showed that the phosphorylation of AMPKThr172, CRTC2 and HDAC5 was stimulated with C3G in HepG2 cells (Figure 1D). Animal experiments showed similar results compared with those found in HepG2 cell line experiments (data not shown). Together, these results demonstrate that C3G suppresses gluconeogenesis-related gene expression through the activation of the AMPK-CRTC2-CREB and AMPK-HDAC5-FOXO1 axes, respectively. FOXO1 may be more important than CREB to the suppression of gluconeogenesis gene expression by C3G.

Next, we performed additional experiments in cells with AMPKα1/2 gene knockdown through siRNA. The knockdown of AMPKα1/2 gene expression negated the effects of C3G on the phosphorylation of AMPK, CRTC2 and HDAC5 (Figure 1E). The knockdown of AMPKα1/2 gene expression also negated the effects of metformin (a positive control) and C3G on the rate of gluconeogenesis but did not affect 8-CPT-cAMP (Figure 1F). Together, these results demonstrate that C3G activates AMPK and inhibits hepatic gluconeogenesis in an AMPK-dependent but protein kinase A-independent manner. C3G inhibits gluconeogenesis, perhaps through the activation of the AMPK-CRTC2-CREB and AMPK-HDAC5-FOXO1 signaling axes and the reduction in gluconeogenesis-related gene expression.

### 3.2. C3G Induces Hepatic Autophagy through the AMPK-mTORC1 Signaling Axis

In the investigation of key metabolites in the livers, C3G dramatically increased the concentrations of branched amino acids and glutathione (Supplementary Table S2). AMPK can activate autophagy pathways and suppress aging; therefore, we next investigated whether C3G induces hepatic autophagy. The activation of hepatic autophagy improved glucose tolerance and slowed aging [30]. Mice fed with HFD were administered vehicle, C3G or fenofibrate (FF) for 6 weeks, and their livers were collected for analysis. HepG2 cells were treated with C3G and analyzed for the experiments.

**Figure 2:**
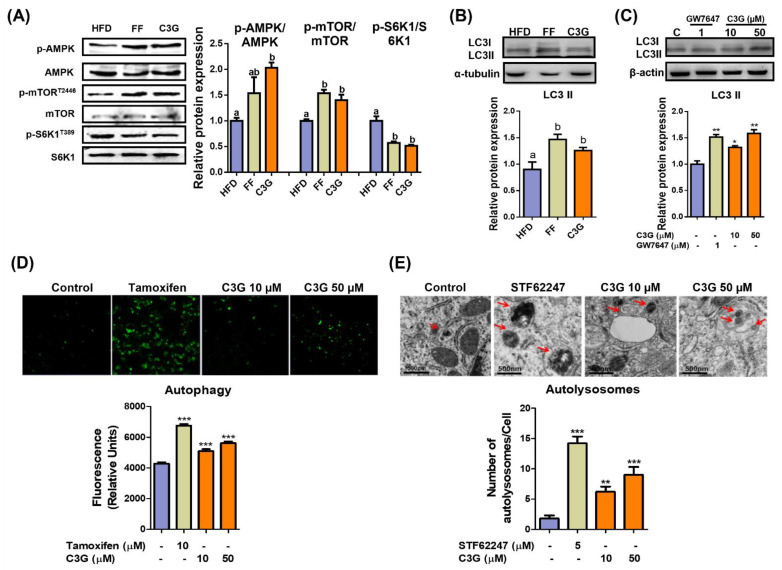
C3G activates hepatic autophagy via the AMPK-mTOR signaling pathway. (**A**) C3G stimulated AMPK, mTOR and S6K1 phosphorylation in the livers of mice in the HFD, fenofibrate (FF) and C3G groups. (**B**,**C**) LC3-II expression in mouse livers and HepG2 cells, respectively. GW7647 is a highly selective PPARα agonist. (**D**) MDC fluorescence staining for autophagy in HepG2 cells treated with tamoxifen and C3G. (**E**) Analysis of hepatic autolysosomes in HepG2 cells treated with STF62247 (a small molecule agonist that induces autophagy, and C3G using TEM). For comparisons between two groups, Student’s *t*-test was performed. *, *p* < 0.05; **, *p* < 0.01; ***, *p* < 0.005 compared with controls.

The phosphorylation levels of AMPKThr172 and mTORC1Thr2446 increased, while phosphorylation of S6K1, an mTORC1 substrate, decreased (Figure 2A), which suggests the suppression of mTORC1 activity and its downstream substrate S6K1 due to AMPK activation. C3G increased the level of LC3-II protein, a marker of autophagosomes, in both the livers and HepG2 cells (Figure 2B,C). The induction of autophagy by C3G was confirmed through TEM and staining with MDC, a fluorescent probe for autolysosomes, in HepG2 cells. MDC staining revealed that C3G significantly increased the number of autolysosomes in HepG2 cells (Figure 2D), and TEM images demonstrated that the number of autolysosomes was significantly elevated in C3G-treated HepG2 cells in vitro (Figure 2E). Together, these results indicate that C3G activates hepatic autophagy through the regulation of the AMPK-mTORC1 signaling axis. The activation of hepatic autophagy may contribute to improvements in insulin sensitivity and aging.

### 3.3. C3G Activates AMPK via Adiponectin Receptor Signaling

**Figure 3:**
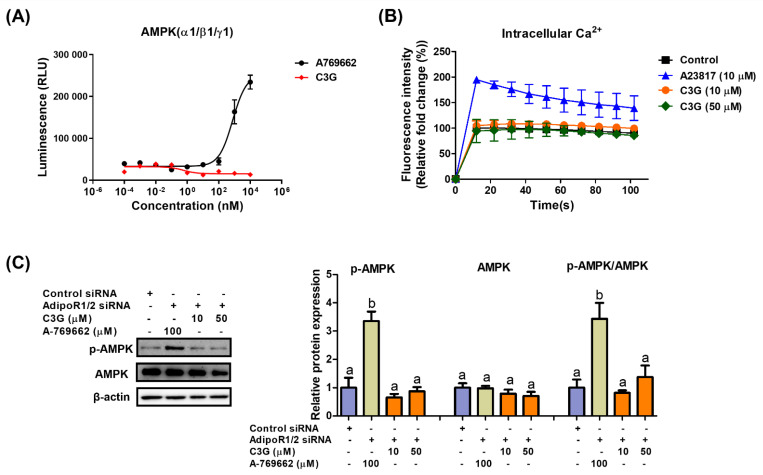
Mechanism through which C3G activates AMPK. (**A**) In vitro AMPK kinase assay. (**B**) Analysis of intracellular calcium in HepG2 cells stimulated with A23817 or C3G. (**C**) AMPK phosphorylation in cells following AdipoR1/2 gene knockdown. For multiple-group comparisons, one-way ANOVA was performed. Different letters indicate significant differences at *p* < 0.05.

We next explored the mechanism of AMPK activation by C3G. AMPK is a heterotrimeric protein including an α catalytic subunit, a β scaffolding subunit and a γ regulatory subunit. The AMPK α1-, β1- and γ1-subunits are ubiquitously expressed in most tissues; therefore, the α1β1γ1 complex is used in AMPK assays to screen AMPK binders [31]. An in vitro kinase assay was performed to investigate the binding of C3G to AMPKαβγ proteins. A-769662 (positive control), an allosteric activator, binds and activates AMPK (Figure 3A). C3G did not bind to AMPK proteins directly and failed to induce AMPK activity in vitro. Adiponectin receptors stimulate AMPK phosphorylation by activating the upstream kinases LKB-1 and CAMKKβ; therefore, we investigated whether C3G activates AMPK via adiponectin receptors. In an intracellular calcium assay, A23817, a calcium ionophore, increased intracellular calcium concentrations, while C3G had no effect on intracellular calcium concentrations (Figure 3B). This finding suggests that C3G does not stimulate AMPK through a calcium-dependent mechanism. The induction of AMPK phosphorylation by C3G was abrogated when adiponectin receptor-1/2 was knocked down using siRNA. These findings suggest that C3G stimulates AMPK via an adiponectin receptor signaling pathway that is independent of intracellular calcium concentration (Figure 3C). Additionally, plasma adiponectin and hepatic AMP levels were elevated in mice fed C3G (Supplementary Table S2). These findings demonstrate that C3G activates AMPK via adiponectin receptor signaling, at least in the liver. Increased intracellular AMP levels and plasma adiponectin may also play significant roles in this process.

### 3.4. C3G Is an Antioxidative Molecule That Induces Cellular Senescence in Hepatocarcinoma Cells

**Figure 4:**
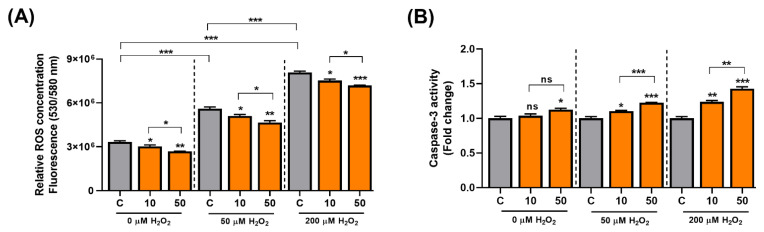
C3G modulates ROS and cell signaling in hepatocarcinoma cells. (**A**) ROS assay. (**B**) 3-caspase activity of HepG2 cells co-treated with 0, 50 and 200 µM H_2_O_2_. The results represent the mean ± SEM (n = 3). For comparisons between two groups, Student’s *t*-test was performed. *, *p* < 0.05; **, *p* < 0.01; ***, *p* < 0.005 compared with controls. C, control (0.1% DMSO); C3G, Cyanidin 3-glucoside; ns, not statistically significant.

C3G activates the AMPK-autophagy axis, and the activation of autophagy can delay aging. ROS cause mitochondrial damage and cellular senescence, and C3G also has potent antioxidative activity. Thus, C3G may suppress carcinogenesis by inducing senescence in cancer cells. To test this possibility, we induced senescence in HepG2 human hepatocarcinoma cells using H_2_O_2_ and examined the antioxidative and anti-cancer effects of C3G. Initially, the antioxidant effect of C3G was then investigated using a fluorometric assay. HepG2 cells were incubated with 0, 50 or 200 µM H_2_O_2_ and co-treated with DMSO (0.1% *v*/*v*, vehicle) or C3G (10 or 50 μM) for 24 h. ROS levels were prominently higher in HepG2 cells incubated with H_2_O_2_ (Figure 4A, grey bars); in contrast, the ROS concentration was significantly reduced in cells co-treated with C3G (Figure 4A; grey versus orange bars). Our results suggested that C3G could decrease the concentration of ROS in cells under oxidative conditions.

Next, since Caspase-3 is a key initiator in the apoptotic-mode of cell death. We investigated the effect of C3G on apoptosis in senescent cells. C3G significantly induced caspase-3 activity in senescence-induced HepG2 cells by 23.7% (10 μM) and 42.8% (50 μM) compared with the controls (Figure 4B). Then, we assessed the effect of C3G on cell proliferation using the MTT assay. The results showed that proliferation was reduced in cells treated with H_2_O_2_ (Supplementary Figure S1, gray bars) and marginally restored with C3G treatment (Supplementary Figure S1, orange bars). The recovery of proliferation was correlated with the ROS reduction caused by C3G. These findings suggest that the removal of intracellular ROS by C3G may contribute to the recovery of proliferation activity.

**Figure 5:**
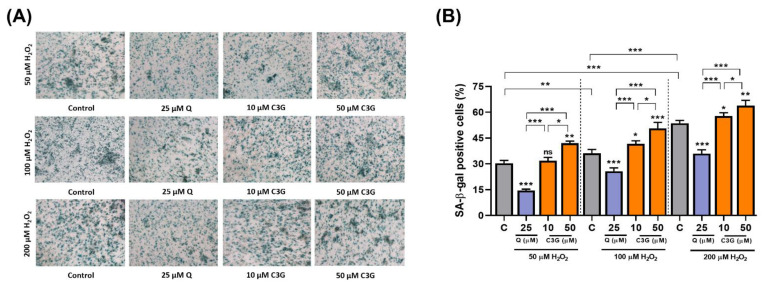
Effect of C3G on anti-cancer activity by inducing senescence in hepatocarcinoma cells. (**A**) SA-β-Gal activity in HepG2 cells treated with 50, 100 and 200 µM H_2_O_2_. Micrographs were obtained with phase contrast microscopy. (**B**) Quantification of SA-β-Gal+ cells cultures shown in (**A**). SA-β-Gal+ cells were scored in 3 fields of at least 450 total cells. The results represent the mean ± SEM (n = 3). For comparisons between two groups, Student’s *t*-test was performed. *, *p* < 0.05; **, *p* < 0.01; ***, *p* < 0.005 compared with controls. C, control (0.1% DMSO); C3G, Cyanidin 3-glucoside; Q. Quercetin; ns, not statistically significant.

In the analysis of senescence-associated β-galactosidase (SA-β-gal) expression, C3G significantly increased SA-β-gal-positive staining by 12.3 ~ 54.8% in H_2_O_2_-treated cells (Figure 5A,B). These results suggest that C3G may achieve anti-cancer activity by inducing senescence in hepatocarcinoma cells.

**Figure 6:**
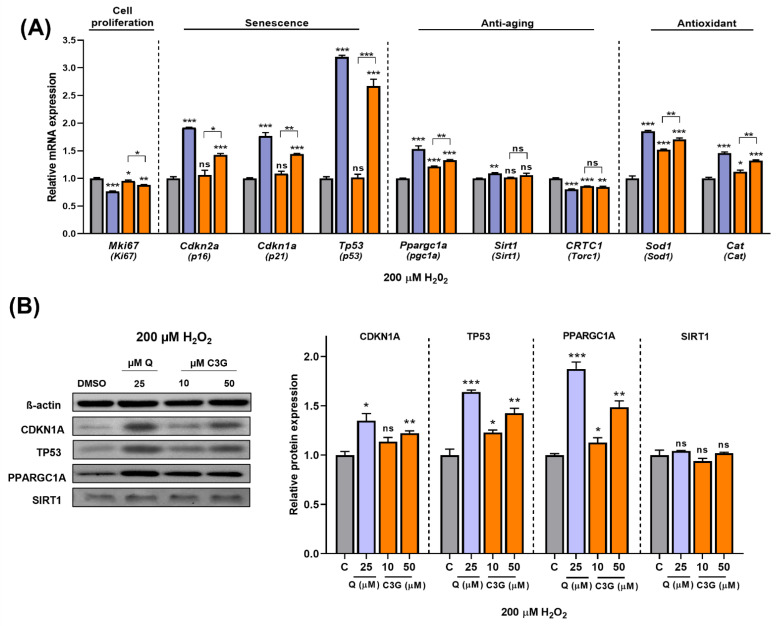
Effect of C3G in cell proliferation, senescence, anti-aging and antioxidant markers in HepG2 after H_2_O_2_-induced senescence. (**A**) RT-qPCR. (**B**) Immunoblotting. The results represent the mean ± SEM (*n* = 3). For comparisons between two groups, Student’s *t*-test was performed. *, *p* < 0.05; **, *p* < 0.01; ***, *p* < 0.005 compared with controls. C, control (0.1% DMSO); C3G, Cyanidin 3-glucoside; Q, quercetin; ns, not statistically significant.

Next, we quantified the expression of key genes related to antioxidant activity and cellular senescence. The expression levels of *Sod1* and *Cat*, antioxidative enzyme genes, were significantly induced by C3G. Cellular antioxidant systems, such as Sod and Cat, can enhance the ability of cells to remove ROS and prevent cellular damage. These findings suggest that C3G induces antioxidant gene expression while also scavenging ROS directly to achieve its potent antioxidant effects. Additionally, the expression of the *Ki67* gene (*Mki67*), a marker of cell proliferation, was significantly suppressed by C3G. The gene expression of the senescence markers *p16*, *p21* and *p53* was significantly induced by C3G (Figure 6A).

We performed further experiments to determine the potential mechanism underlying these results. As shown in Figure 6B, given stress-induced senescence triggered by exposure to 200 µM H_2_O_2_, co-treatment with C3G marginally rescued the loss of SIRT1 protein. Likewise, co-treatment with C3G significantly upregulated pgc1α, p21 and p53 expression in a dose-dependent manner. These results indicated that C3G regulates the proliferation and antioxidant activity of HepG2 cells by increasing the expression of key genes and proteins involved in senescence and antioxidant activity in hepatocarcinoma cells.

## 4. Discussion

C3G is a potent phytochemical that is abundant in plant-based foods, such as leafy vegetables, teas, berries and colored grains [22,23,27]. We investigated the biological mechanism of C3G activity in gluconeogenesis and cancer cell senescence in liver and hepatocytes. C3G activated AMPK activity, and then AMPK phosphorylated and inactivated the CRTC2 and HDAC5 coactivators, which are required for the expression of *Pepck* and *G6pc*, two key genes in gluconeogenesis. Thus, the reduced expression of *Pepck* and *G6pc* suppressed glucose production in mouse livers and cultured hepatocytes.

C3G induced AMPK phosphorylation in both cultured hepatocytes and mouse livers, and AMPK activation by C3G suppressed hepatic gluconeogenesis by inhibiting FOXO1 and CREB activity, thereby subsequently suppressing the expression of these gluconeogenic genes. Phospho-CREB and its coactivator, CREB-regulated transcription coactivator 2 (CRTC2), bind to the promoter sequence TGACGTCA to act as a transcriptional activator of gluconeogenic enzymes such as PC, PEPCK, FBPase-1 and G6Pase [32]. During fasting, the activity of CRTC2-CREB is highest in the nucleus. However, in the postprandial state, under the influence of insulin, CRTC2 undergoes rapid phosphorylation, resulting in its localization to the cytoplasm and the transcriptional repression of its target genes [33]. Similar results have been found for transcription factors belonging to the FoxO family, among which FoxO1 is most highly expressed [34]. FoxO1 binds to HDAC5 to promote the transcriptional expression of the *Pepck* and *G6Pase* genes. AMPK phosphorylates FoxO1, rendering it inactive and thereby preventing its translocation from cytoplasm to nucleus [35]. These activities of C3G were abrogated in cells in which the AMPKα1/2 gene was knocked down, demonstrating that the hypoglycemic effect of C3G is solely due to AMPK activation.

In line with recent findings, we previously demonstrate the mechanism by which C3G, regulates energy metabolism and insulin sensitivity. The oral administration of C3G reduced hepatic and plasma triglyceride levels and adiposity, and improved glucose tolerance in mice fed high-fat diet. While hepatic metabolomic analysis revealed that C3G shifted metabolite profiles towards fatty acid oxidation and ketogenesis [27]. Similarly, C3G increased glucose uptake in HepG2 cells and induced the rate of hepatic fatty acid oxidation.

AMPK activation by C3G suppressed mTORC1 and increased the autophagy pathway, thereby delaying the cellular aging process. Rapamycin and its analogs that primarily inhibit mTORC1 have been reported to exhibit the oncogenic suppression of cellular growth and to reverse metabolic disorders, such as insulin resistance and hepatosteatosis, that arise due to persistent mTORC1 activation [36,37]. In parallel, AMPK activators, such as metformin and A-769662, have been found to mitigate tumorigenesis in mouse cancer models. This effect is due to the involvement of LKB1, a major upstream kinase of AMPK. Many tumors, especially nonsmall cell lung cancer, occur due to a deficiency of the LKB1 gene. Experimental studies have confirmed that AMPK acts as a mediator of LKB1, suppressing tumorigenesis [38,39]. Moreover, AMPK induces autophagy (mitophagy, lipophagy and glycophagy) by phosphorylating two kinase complexes, ULK1 and PI3CK/VP34 [40,41]. In the present study, C3G was found to stimulate the hepatic autophagy pathway via the activation of the AMPK-mTORC1-S6K signaling axis. C3G could successfully coordinate the LC3-II-mediated formation of autophagosomes in cultured liver cells.

In the investigation of the mechanism of action of C3G in AMPK activation, C3G did not directly interact with the subunits of AMPKα1β1γ1 and did not affect the calcium concentration; however, AMPK activation by C3G was negated in cells with adiponectin receptor 1/2 gene knockdown. These findings suggest that C3G may function as a ligand of adiponectin receptors, stimulating AMPK activity.

To elucidate the mechanism of AMPK activation by C3G, we examined whether C3G binds directly to the AMPK protein or stimulates AMPK activities indirectly. AMPKα1β1γ1 is a major AMPK isoform, and the in vitro AMPK kinase assay demonstrated that C3G did not induce AMPK activity. Therefore, we suggest that C3G is neither a direct binder nor an allosteric modulator of AMPK. We next sought to investigate the indirect mechanism through which C3G activates hepatic AMPK. Hepatic AMP and plasma adiponectin levels were significantly elevated in HFD mice treated with C3G (Supplementary Table S2 and Figure 3). Elevated intracellular AMP (or AMP/ATP ratio) stimulates AMPK activity. The reported half-maximal effect of AMP on AMPK activation (EC50) is 2.6 μM in the absence of ATP [42], and thus the intracellular AMP level in hepatocytes (21.3 μM) is sufficient to activate AMPK in the presence of low ATP concentrations (2.8 μM; Supplementary Table S2). Moreover, adiponectin activates AMPK via adiponectin receptor (AdipoR1/R2) signaling pathways. C3G may directly stimulate AdipoR1/R2 signaling pathways to achieve AMPK activation. The results of this study demonstrated that the knockdown of both AdipoR1/R2 using siRNA abrogated AMPK phosphorylation by C3G in cultured hepatocytes. These results indicate that AMPK phosphorylation by C3G is cell specific, and that the activation of AMPK by C3G is induced, at least in part, by direct adiponectin receptor activation. AdipoR1/R2 stimulate distinct downstream signaling cascades; AdipoR1 stimulates the LKB1-AMPK axis, while AdipoR2 induces an increase in intracellular calcium levels, which enhances the CAMKKβ-AMPK axis [43].

Adipokines are fat-derived hormones secreted by white adipose tissue. Adipokines affect insulin sensitivity by modulating proteins associated with insulin signaling, glucose and lipid homeostasis. Due to their antiglycemic and lipid lowering properties, adipokines have received increasing attention for the pharmacological treatment of metabolic disorders, such as hyperlipidemia, cardiovascular diseases and diabetes [44,45]. Plasma adiponectin levels decrease sharply in obese patients with fatty liver disease [46]. Although ubiquitous, AdipoR1 and AdipoR2 are most abundantly expressed in the skeletal muscle and liver, respectively. They belong to the G protein-coupled receptor (GPCR) family, with their N terminus located intracellularly, while the C terminus is outside the cell [47]. Consistent with the AMPK-adiponectin hypothesis, it was reported that elevated levels of hepatic AMPK were primarily responsible for the decreased gluconeogenic activities of PCK and G6Pase enzyme in adiponectin-treated mice [48,49]. It is possible that some of the effects of adiponectin in the liver are also mediated by the central actions of adiponectin and, therefore, independent of the liver LKB1/AMPK/CRTC2 pathway [49].

Anthocyanins are potent antioxidants, and the protective effect of anthocyanins against oxidative stress-induced damage is promising, as shown using in cultured cells and in vivo models [20,25,26]. Likewise, we sought to examine the antioxidative effects of C3G, which can influence cancer cell senescence. H_2_O_2_ induced ROS, which led to oxidative stress in HepG2 hepatocarcinoma cells, and the effect was reduced by C3G, confirming a potent antioxidative effect of C3G. C3G induced apoptosis, as assessed based on caspase-3 activity, in HepG2 cells, suggesting anti-cancer activity. The suppression of cell proliferation is one of the prominent therapeutic strategies employed for cancer treatment, and the induction of cellular senescence could suppress cancer development, migration and metastasis. Therefore, we investigated whether C3G affects senescence in HepG2 hepatocarcinoma cells. The results demonstrated that C3G reduced senescence-associated β-galactosidase, P16, P21 and P53 and increased the cell proliferation marker *Ki-67*. These results demonstrate that C3G exhibits antioxidant and anti-cancer activities through the induction of cellular senescence and apoptosis. Supplementary Figure S2 shows a schematic representation of the several potential molecular pathways that are involved in cellular senescence and how C3G presumably regulates cell fate in an AMPK/p53-dependent manner.

Some questions remain unanswered after this study. We were unable to determine whether C3G binds directly to AdipoR1/2 proteins, although we confirmed that AMPK phosphorylation by C3G was abrogated in cells with AdipoR1/2 gene knockdown. C3G directly initiates AdipoR1/2 signaling while also increasing plasma adiponectin concentrations. In our previous study, C3G induced adiponectin gene expression and protein secretion in cultured adipocytes [27]. As adiponectin also stimulates adipoR1/2 signaling, C3G may activate adipoR1/2 signaling through two mechanisms, direct activation and plasma adiponectin induction; however, we were unable to quantify the individual contributions of these two processes. To quantify their contributions, complex animal experiments including adiponectin- and adipoR1/2-deficient mice will be required. Metformin was previously thought to inhibit the activity of transcription factors associated with gluconeogenesis through the activation of AMPKs. However, scientists later determined that metformin could exhibit its pharmacological properties even in AMPKα knockout or LKB1 knockout mouse models [49]. Moreover, AMPK activation by AdipoR1 regulated cellular lipid and glucose metabolism by activating fatty acid oxidation and autophagy and suppressing hepatic gluconeogenesis [50]. By using gene deletion experiment, Miller et al. demonstrated that LKB1- and AMPK-dependent and independent signaling pathways may exist in vivo [49,51]. In cultured cells, C3G activated AMPK through the adiponectin receptor signaling pathway, involving several targets; thus, we do not rule out the possibility that C3G activates adipoR1/2 signaling in vivo as well. C3G is metabolized rapidly after intestinal absorption; however, it has been reported that the concentration of C3G is substantially high in vivo [27,52]. Therefore, further research is required to provide evidence of the molecular mechanism in vivo.

## 5. Conclusions

Together, our results demonstrate that C3G, a natural antioxidant, has multiple biological activities in hepatocytes. C3G suppressed hepatic glucose production by AMPK activation. The mechanism of action of C3G in AMPK activation is multifactorial. Increased plasma adiponectin levels, an increased hepatic AMP-to-ATP ratio and the activation of the adiponectin receptor signaling pathway collectively activate AMPK. C3G is also able to activate AMPK, improve glucose tolerance and induce hepatic autophagy in HFD-fed obese mice. C3G induced autophagy, which may delay the aging process and improve cellular health. Finally, C3G showed antioxidative and anti-cancer effects through the induction of senescence in hepatocarcinoma cells.

## Data Availability

The study did not report any data. These are available upon reasonable request.

## Supplementary Materials

The following are available online at https://www.mdpi.com/article/10.3390/antiox11040749/s1, Table S1: List of chemical and/or reagents used for the present study. Table S2: Major liver metabolites from mice fed a HFD and C3G for 8 weeks using CE-MS and GC-TOF-MS. Figure S1: C3G modulates ROS and cell signalling in hepatocarcinoma cells. Figure S2: Several potential molecular pathways are involved in cellular senescence and C3G presumably regulates cell fate in an AMPK/p53-dependent manner.

## References

[1] Seo E., Kang H., Choi H., Choi W., Jun H.S. (2019). Reactive oxygen species-induced changes in glucose and lipid metabolism contribute to the accumulation of cholesterol in the liver during aging. Aging Cell.

[2] Reczek C.R., Chandel N.S. (2017). The two faces of reactive oxygen species in cancer. Annu. Rev. Cancer Biol..

[3] Fitzgerald A.L., Osman A.A., Xie T.X., Patel A., Skinner H., Sandulache V., Myers J.N. (2015). Reactive oxygen species and p21(Waf1/Cip1) are both essential for p53-mediated senescence of head and neck cancer cells. Cell Death Dis..

[4] Davalli P., Mitic T., Caporali A., Lauriola A., D’Arca D. (2016). ROS, cell senescence, and novel molecular mechanisms in aging and age-related diseases. Oxid. Med. Cell. Longev..

[5] Ahmed E.K., Rogowska-Wrzesinska A., Roepstorff P., Bulteau A.L., Friguet B. (2010). Protein modification and replicative senescence of WI-38 human embryonic fibroblasts. Aging Cell.

[6] Lawless C., Jurk D., Gillespie C.S., Shanley D., Saretzki G., von Zglinicki T., Passos J.F. (2012). A stochastic step model of replicative senescence explains ROS production rate in ageing cell populations. PLoS ONE.

[7] Vigneron A., Vousden K.H. (2010). p53, ROS and senescence in the control of aging. Aging.

[8] Passos J.F., Nelson G., Wang C., Richter T., Simillion C., Proctor C.J., Miwa S., Olijslagers S., Hallinan J., Wipat A. (2010). Feedback between p21 and reactive oxygen production is necessary for cell senescence. Mol. Syst. Biol..

[9] Malavolta M., Costarelli L., Giacconi R., Piacenza F., Basso A., Pierpaoli E., Marchegiani F., Cardelli M., Provinciali M., Mocchegiani E. (2014). Modulators of cellular senescence: Mechanisms, promises, and challenges from in vitro studies with dietary bioactive compounds. Nutr. Res..

[10] Yan L.-J. (2014). Positive oxidative stress in aging and aging-related disease tolerance. Redox Biol..

[11] Palsamy P., Subramanian S. (2010). Ameliorative potential of resveratrol on proinflammatory cytokines, hyperglycemia mediated oxidative stress, and pancreatic beta-cell dysfunction in streptozotocin-nicotinamide-induced diabetic rats. J. Cell. Physiol..

[12] Gibellini L., Pinti M., Nasi M., De Biasi S., Roat E., Bertoncelli L., Cossarizza A. (2010). Interfering with ROS metabolism in cancer cells: The potential role of quercetin. Cancers.

[13] Yılmaz Göler A., Biçim G., Toprak K., Yılmaz B., Yalçın A., Milisav I. (2021). Hydrogen Peroxide and Quercetin Induced Changes on Cell Viability, Apoptosis and Oxidative Stress in HepG2 Cells. Curr. Nutraceuticals.

[14] Acquaviva R., Tomasello B., Di Giacomo C., Santangelo R., La Mantia A., Naletova I., Sarpietro M.G., Castelli F., Malfa G.A. (2021). Protocatechuic Acid, a Simple Plant Secondary Metabolite, Induced Apoptosis by Promoting Oxidative Stress through HO-1 Downregulation and p21 Upregulation in Colon Cancer Cells. Biomolecules.

[15] Badiola I., Santaolalla F., Garcia-Gallastegui P., Unda F., Ibarretxe G. (2015). Biomolecular bases of the senescence process and cancer. A new approach to oncological treatment linked to ageing. Ageing Res. Rev..

[16] Vurusaner B., Poli G., Basaga H. (2012). Tumor suppressor genes and ROS: Complex networks of interactions. Free Radic. Biol. Med..

[17] Alers S., Loffler A.S., Wesselborg S., Stork B. (2012). Role of AMPK-mTOR-Ulk1/2 in the Regulation of Autophagy: Cross Talk, Shortcuts, and Feedbacks. Mol. Cell. Biol..

[18] Lamming D.W., Ye L., Katajisto P., Goncalves M.D., Saitoh M., Stevens D.M., Davis J.G., Salmon A.B., Richardson A., Ahima R.S. (2012). Rapamycin-induced insulin resistance is mediated by mTORC2 loss and uncoupled from longevity. Science.

[19] Selman C., Tullet J.M.A., Wieser D., Irvine E., Lingard S.J., Choudhury A.I., Claret M., Al-Qassab H., Carmignac D., Ramadani F. (2009). Ribosomal protein S6 kinase 1 signaling regulates mammalian life span. Science.

[20] Joshi T., Singh A.K., Haratipour P., Sah A.N., Pandey A.K., Naseri R., Juyal V., Farzaei M.H. (2019). Targeting AMPK signaling pathway by natural products for treatment of diabetes mellitus and its complications. J. Cell. Physiol..

[21] Salomone F., Godos J., Zelber-Sagi S. (2016). Natural antioxidants for non-alcoholic fatty liver disease: Molecular targets and clinical perspectives. Liver Int..

[22] Chen J., Xu B., Sun J., Jiang X., Bai W. (2021). Anthocyanin supplement as a dietary strategy in cancer prevention and management: A comprehensive review. Crit. Rev. Food Sci. Nutr..

[23] He J., Giusti M.M. (2010). Anthocyanins: Natural colorants with health-promoting properties. Annu. Rev. Food Sci. Technol..

[24] Mannino G., Gentile C., Ertani A., Serio G., Bertea C.M. (2021). Anthocyanins: Biosynthesis, distribution, ecological role, and use of biostimulants to increase their content in plant foods—A review. Agriculture.

[25] Gowd V., Jia Z., Chen W. (2017). Anthocyanins as promising molecules and dietary bioactive components against diabetes–A review of recent advances. Trends Food Sci. Technol..

[26] Zhu W., Jia Q., Wang Y., Zhang Y., Xia M. (2012). The anthocyanin cyanidin-3-O-β-glucoside, a flavonoid, increases hepatic glutathione synthesis and protects hepatocytes against reactive oxygen species during hyperglycemia: Involvement of a cAMP–PKA-dependent signaling pathway. Free Radic. Biol. Med..

[27] Jia Y., Wu C., Kim Y.-S., Yang S.O., Kim Y., Kim J.-S., Jeong M.-Y., Lee J.H., Kim B., Lee S. (2020). A dietary anthocyanin cyanidin-3-O-glucoside binds to PPARs to regulate glucose metabolism and insulin sensitivity in mice. Commun. Biol..

[28] Wu C., Hwang S.H., Jia Y., Choi J., Kim Y.J., Choi D., Pathiraja D., Choi I.G., Koo S.H., Lee S.J. (2017). Olfactory receptor 544 reduces adiposity by steering fuel preference toward fats. J. Clin. Investig..

[29] Steinberg G.R., Carling D. (2019). AMP-activated protein kinase: The current landscape for drug development. Nat. Rev. Drug Discov..

[30] Bechmann L.P., Hannivoort R.A., Gerken G., Hotamisligil G.S., Trauner M., Canbay A. (2012). The interaction of hepatic lipid and glucose metabolism in liver diseases. J. Hepatol..

[31] Herzig S., Shaw R.J. (2018). AMPK: Guardian of metabolism and mitochondrial homeostasis. Nat. Rev. Mol. Cell. Biol..

[32] Mayr B., Montminy M. (2001). Transcriptional regulation by the phosphorylation-dependent factor CREB. Nat. Rev. Mol. Cell. Biol..

[33] Dentin R., Liu Y., Koo S.-H., Hedrick S., Vargas T., Heredia J., Yates J., Montminy M. (2007). Insulin modulates gluconeogenesis by inhibition of the coactivator TORC2. Nature.

[34] Quinn P., Yeagley D. (2005). Insulin regulation of PEPCK gene expression: A model for rapid and reversible modulation. Endocr Metab Immune Disord. Drug Targets.

[35] Housley M.P., Udeshi N.D., Rodgers J.T., Shabanowitz J., Puigserver P., Hunt D.F., Hart G.W. (2009). A PGC-1α-O-GlcNAc transferase complex regulates FoxO transcription factor activity in response to glucose. J. Biol. Chem..

[36] López-Otín C., Blasco M.A., Partridge L., Serrano M., Kroemer G. (2013). The hallmarks of aging. Cell.

[37] Ueno T., Komatsu M. (2017). Autophagy in the liver: Functions in health and disease. Nat. Rev. Gastroenterol. Hepatol..

[38] Alessi D.R., Sakamoto K., Bayascas J.R. (2006). LKB1-dependent signaling pathways. Annu. Rev. Biochem..

[39] Hemminki A. (1999). The molecular basis and clinical aspects of Peutz-Jeghers syndrome. Cell. Mol. Life Sci..

[40] Kim J., Kim Y.C., Fang C., Russell R.C., Kim J.H., Fan W., Liu R., Zhong Q., Guan K.L. (2013). Differential regulation of distinct Vps34 complexes by AMPK in nutrient stress and autophagy. Cell.

[41] Kim J., Kundu M., Viollet B., Guan K.L. (2011). AMPK and mTOR regulate autophagy through direct phosphorylation of Ulk1. Nat. Cell Biol..

[42] Gowans G.J., Hawley S.A., Ross F.A., Hardie D.G. (2013). AMP is a true physiological regulator of AMP-activated protein kinase by both allosteric activation and enhancing net phosphorylation. Cell Metab..

[43] Zha D., Wu X., Gao P. (2017). Adiponectin and its receptors in diabetic kidney disease: Molecular mechanisms and clinical potential. Endocrinology.

[44] Kadowaki T., Yamauchi T. (2005). Adiponectin and adiponectin receptors. Endocr. Rev..

[45] Kadowaki T., Yamauchi T., Kubota N., Hara K., Ueki K., Tobe K. (2006). Adiponectin and adiponectin receptors in insulin resistance, diabetes, and the metabolic syndrome. J. Clin. Investig..

[46] Belfort R., Harrison S.A., Brown K., Darland C., Finch J., Hardies J., Balas B., Gastaldelli A., Tio F., Pulcini J. (2006). A placebo-controlled trial of pioglitazone in subjects with nonalcoholic steatohepatitis. N. Engl. J. Med..

[47] Yamauchi T., Kamon J., Ito Y., Tsuchida A., Yokomizo T., Kita S., Sugiyama T., Miyagishi M., Hara K., Tsunoda M. (2003). Cloning of adiponectin receptors that mediate antidiabetic metabolic effects. Nature.

[48] Combs T.P., Pajvani U.B., Berg A.H., Lin Y., Jelicks L.A., Laplante M., Nawrocki A.R., Rajala M.W., Parlow A.F., Cheeseboro L. (2004). A transgenic mouse with a deletion in the collagenous domain of adiponectin displays elevated circulating adiponectin and improved insulin sensitivity. Endocrinology.

[49] Miller R.A., Chu Q., Le Lay J., Scherer P.E., Ahima R.S., Kaestner K.H., Foretz M., Viollet B., Birnbaum M.J. (2011). Adiponectin suppresses gluconeogenic gene expression in mouse hepatocytes independent of LKB1-AMPK signaling. J. Clin. Investig..

[50] Fryer L.G., Parbu-Patel A., Carling D. (2002). The anti-diabetic drugs rosiglitazone and metformin stimulate AMP-activated protein kinase through distinct signaling pathways. J. Biol. Chem..

[51] Miller R.A., Birnbaum M.J. (2010). An energetic tale of AMPK-independent effects of metformin. J. Clin. Investig..

[52] Yang Z., Huang T., Li P., Ai J., Liu J., Bai W., Tian L. (2021). Dietary fiber modulates the fermentation patterns of cyanidin-3-O-glucoside in a fiber-type dependent manner. Foods.

