# Supplementary material for: Mechanism of Action of Cyanidin 3-O-Glucoside in Gluconeogenesis and Oxidative Stress-Induced Cancer Cell Senescence
Source: Antioxidants (Basel). 2022 Apr 9;11(4):749. doi: 10.3390/antiox11040749 (PMC9029247; doi:10.3390/antiox11040749)
Supplement: Supplementary file 1 [file antioxidants-11-00749-s001.zip › antioxidants-1669296-supplementary.pdf]

# Mechanism of action of cyanidin 3-*O*-glucoside in gluconeogenesis and oxidative stress-induced cancer cell senescence

**Yaoyao Jia[1,†], Chunyan Wu[1,†], Adriana Rivera-Piza[1,†],Yeon-Ji Kim[1], Ji Hae Lee[1], Sung-Joon Lee[1,2,*]**

[1] Department of Biotechnology, College of Life Science and Biotechnology. Korea University, Seoul 02841 Korea
[2] Department of Food Bioscience and Technology, College of Life Sciences and Biotechnology, Korea University, Seoul 02841 Republic of Korea
† These authors equally contributed to this study.
* Correspondence: junelee@korea.ac.kr; Department of Biotechnology, College of Life Science and Biotechnology. Korea University, Seoul 02841 Korea; Department of Food Bioscience and Technology, College of Life Sciences and Biotechnology, Korea University, Seoul 02841 Republic of Korea; Tel.: +82-2-3290-302

## Supplementary materials

**Table S1.** List of chemical and/or reagents used for the present study.

| Chemical Reagents | Source | Catalogue # |
|---|---|---|
| Dulbecco's modified Eagle's medium (DMEM) | HyClone (Logan, UT, USA) | SH30243.01 |
| Foetal bovine serum (FBS) | HyClone (Logan, UT, USA) | SH30084.03 |
| Penicillin/streptomycin (PEST) | Welgene Inc. | SV30010 |
| Cyanidin-3-O-glucoside (C3G) | Tokiwa Phytochemical Co. (Japan) | P21021 |
| Troglitazone (TT) | Sigma Aldrich (St. Louis, MO) | T2573 |
| GW7647 | Cayman Chemical (Michigan, USA) | 10008613 |
| GW0742 | Cayman Chemical (Michigan, USA) | 10006798 |
| 8-CPT-cAMP | Cayman Chemical (Michigan, USA) | 12011 |
| Compound C | Sigma Aldrich (St. Louis, MO) | 171260 |
| RNAiso Plus | Takara (Otsu, Japan) | 9109 |
| Thunderbird™ SYBR® qPCR Mix | Toyobo (Osaka, Japan) | QPS-201 |
| Triglycerides | Cobas111 (Roche, Switzerland) | 46577594 |
| Cholesterol | Cobas111 (Roche, Switzerland) | 04718917 |
| Aspartate aminotransferase | Cobas111 (Roche, Switzerland) | 04657543 |
| Alanine aminotransferase | Cobas111 (Roche, Switzerland) | 04718569 |
| HDL-cholesterol | Cobas111 (Roche, Switzerland) | 07528604 |
| LDL-cholesterol | Cobas111 (Roche, Switzerland) | 07005806 |
| Control siRNA | Santa Cruz (CA, USA) | sc-37007 |
| **Antibodies** | **Source** | **Catalogue #** |
| PDK4 | Santa Cruz (CA, USA) | sc-14495 |
| PDH1$\alpha$ | Abcam (Cambridge, UK) | ab110330 |
| p-PDH1$\alpha$ | Abcam (Cambridge, UK) | ab92696 |
| AMPK | Santa Cruz (CA, USA) | sc-74461 |
| p-AMPK | Santa Cruz (CA, USA) | sc-33524 |
| PPARGC1 | Santa Cruz (CA, USA) | sc-74504 |
| TP53 | Santa Cruz (CA, USA) | sc-126 |
| SIRT1 | Santa Cruz (CA, USA) | sc-74504 |
| CDKN1A | Santa Cruz (CA, USA) | sc-6246 |
| $\beta$-Actin | Santa Cruz (CA, USA) | sc-47778 |
| Secondary antibodies (anti-mouse) | Santa Cruz (CA, USA) | 31430 |
| **Cell Lines** | **Source** | **Catalogue #** |
| HepG2 (Human hepatocellular carcinoma) | Korean Cell Line Bank | 88065 |
| **Mice** | **Source** | **Catalogue #** |

| | | |
|---|---|---|
| C57BL/6J | Samtako Co. (Kyunggido, Korea) | C57BL/6JTacSam |
| PPARα-deficient | Taconic (Hudson, NY, USA) | 1640 |
| **Assay Kits** | **Source** | **Catalogue #** |
| Adiponectin | Abcam (Cambridge, UK) | ab108785 |
| Insulin | Alpco (NH, USA) | 80-INSMSU-E01 |
| Malonyl-CoA | Cusabio Biotech Co. (TX, USA) | CSB-E12896m |
| β-Hydroxybutyrate | Cayman (MI, USA) | 700190 |
| LanthaScreen™ TR-FRET | Invitrogen (Carlsbad, CA, USA) | A15878 |
| FGF21 | Merck Millipore | EZRMFGF21-26K |
| Glycogen | Abcam (Cambridge, UK) | ab169558 |

**Table S2.** Major liver metabolites from mice fed a HFD and C3G for 8 weeks using CE-MS and GC-TOF-MS.

| **Metabolites** | **CE-MS relative peak area (mean ± SD)[1]** | |
|---|---|---|
| | **HFD[2]** | **C3G[3]** |
| *Purine metabolism* | | |
| ATP | 14.201±0.661 | 11.847±0.414* |
| ADP | 11.182±0.329 | 10.728±0.201 |
| AMP | 12.594±0.443 | 17.245±0.905* |
| Adenine | 6.315±0.508 | 8.433±0.325* |
| Adenosine | 3.470±0.278 | 3.038±0.030 |
| *Glycolysis* | | |
| Glucose 6-phosphate | 25.445±0.567 | 29.021±0.610* |
| Fructose 6-phosphate | 8.591±0.176 | 10.051±0.273* |
| Fructose 1,6-diphosphate | 0.653±0.067 | 0.635±0.100 |
| Dihydroxyacetone phosphate | 8.268±0.338 | 11.394±0.098* |
| 3-Phosphoglyceric acid | 70.567±1.293 | 41.054±0.929* |
| 2-Phosphoglyceric acid | 7.956±0.195 | 4.822±0.134* |
| Phosphoenolpyruvic acid | 17.815±0.337 | 10.217±0.095* |
| Pyruvic acid | 54.875±1.452 | 30.684±2.973* |
| Lactic acid | 10140.602±101.363 | 9024.705±350.355* |
| Fructose 1-phosphate | 1.944±0.776 | 1.348±0.737* |
| Glycerol 3-phosphate | 230.470±6.285 | 779.513±25.265* |
| 2,3-Diphosphoglyceric acid | 4.747±0.057 | 0.409±0.024* |
| *TCA cycle* | | |
| Acetyl CoA | 0.032±0.013 | 0.027±0.010 |
| CoA | 0.412±0.012 | 0.505±0.028* |
| Citric acid | 3.995±0.455 | N.D. |
| α-Ketoglutaric acid | 12.884±0.588 | 8.084±1.471* |
| Succinic acid | 62.239±1.267 | 135.582±4.424* |
| Fumaric acid | 700.874±9.309 | 599.059±16.568* |
| Malic acid | 2106.790±28.503 | 1795.026±79.442* |
| *Pentose phosphate pathway* | | |
| 6-Phosphogluconic acid | 50.175±1.396 | 60.930±1.607* |
| Ribose 5-phosphate | 1.061±0.061 | 1.104±0.122 |
| UDP-glucose | 0.781±0.029 | 0.644±0.051* |
| ADP-ribose | 0.468±0.021 | 0.429±0.024 |
| Galactose 1-phosphate | 51.098±0.153 | 46.520±1.665* |
| Glucose 1-phosphate | 2.141±0.789 | 2.238±1.271 |
| Ribose 1-phosphate | 20.622±1.004 | 26.839±0.737* |

| | | |
|---|---|---|
| *Glutathione metabolism* | | |
| Glutathione (GSH) | 76.586±3.408 | 158.868±5.457* |
| Glutathione (GSSG) | 1633.081±35.013 | 1197.879±26.676* |
| *S*-Adenosylhomocysteine | 10.261±0.119 | 12.041±0.695 |
| Cystathionine | 35.019±1.206 | 27.087±1.050* |
| Homoserine | 3.362±0.726 | 6.506±1.732 |
| Thr | 4798.143±132.978 | 5719.924±160.113* |
| *Carnitine and choline metabolism* | | |
| Carnitine | 300.006±5.024 | 327.691±1.952* |
| Choline | 2150.539±113.030 | 2923.430±123.61* |
| Betaine aldehyde | 413.371±8.955 | 475.428±9.235* |
| Betaine | 764.813±8.778 | 776.519±15.539 |
| Folic acid | 0.238±0.022 | 0.197±0.008* |

[1] Values are the means of three replicates for the peak area relative to the internal standard ± standard deviation (SD). *, $P<0.05$; [2] High-fat diet 45%; [4] High-fat diet 45% + cyanidin 3-*O*-β–D-glucoside.

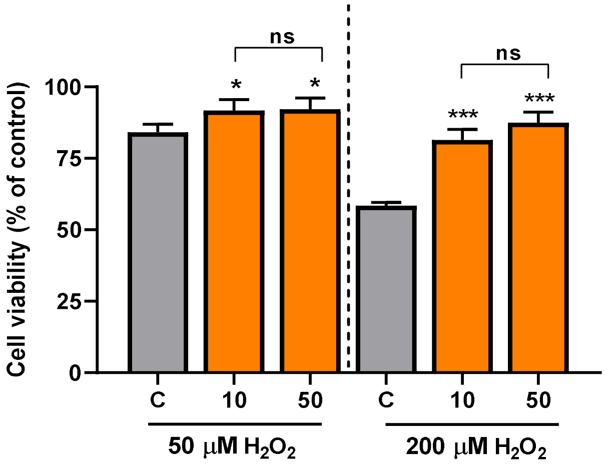


**Figure S1.** C3G modulates ROS and cell signalling in hepatocarcinoma cells. A) Effect of C3G on the viability of HepG2 cells co-treated with 50 and 200 μM $H_2O_2$ for 24h. The results represent the mean ± SEM (n=3). For comparisons between two groups, Student's t-test was performed. *, $P < 0.05$; **, $P < 0.01$; ***, $P < 0.005$ compared with controls. C, control (0.1% DMSO); C3G, Cyanidin 3-glucoside; ns, not statistically significant.

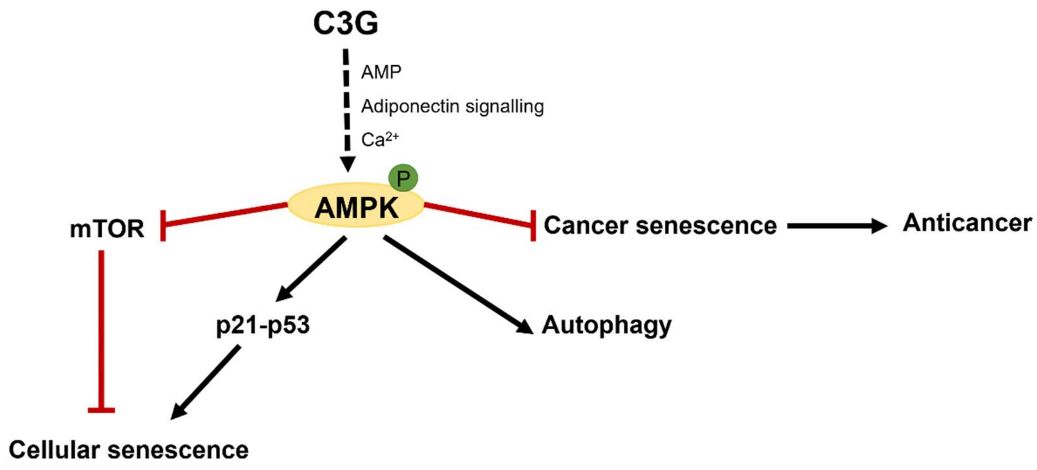


**Figure S2.** Several potential molecular pathways are involved in cellular senescence and C3G presumably regulates cell fate in an AMPK/p53-dependent manner.
